# Effects of Feeding Frequency on the Lying Behavior of Dairy Cows in a Loose Housing with Automatic Feeding and Milking System

**DOI:** 10.3390/ani9040121

**Published:** 2019-03-28

**Authors:** Gabriele Mattachini, Johanna Pompe, Alberto Finzi, Emanuela Tullo, Elisabetta Riva, Giorgio Provolo

**Affiliations:** 1 Department of Agricultural and Environmental Sciences, Università degli Studi di Milano, 20133 Milano, Italy; alberto.finzi@unimi.it (A.F.); elisabetta.riva@unimi.it (E.R.); giorgio.provolo@unimi.it (G.P.); 2 Farm Technology Group, Wageningen University & Research, Droevendaalsesteeg 1, 6708PB Wageningen, The Netherlands; hanneke.pompe@gmail.com; 3 Department of Environmental Science and Policy, Università degli Studi di Milano, 20133 Milano, Italy; emanuela.tullo@unimi.it

**Keywords:** feed delivery frequency, lying behavior, dairy cow, automatic feeding system (AFS), automatic milking system (AMS)

## Abstract

**Simple Summary:**

Feeding management in modern dairy farms is becoming increasingly important economically and technologically as well as concern for the comfort and welfare of dairy cows. Automatic feeding systems enable more frequent delivery of fresh feed to dairy cows compared to conventional feeding systems and, in combination with automatic milking systems, provide cows more freedom to determine individual critical behavioral activities (feeding, lying, and milking). This study examined the effects of feed delivery frequency on behavior of lactating dairy cows in an automatic feeding and milking system. The results highlight a potential negative effect of high frequency (11 times per day) feed delivery compared to low frequency (six times per day) on lying behavior. This indication can improve feeding management and the productivity and welfare of lactating dairy cows.

**Abstract:**

Management systems in modern dairy farms is an important issue in relation to animal comfort and welfare. The objective of this study was to determine the effect of feed delivery frequency on the behavior patterns, visits to an automatic milking system (AMS) and on milk production of lactating dairy cows. The study was conducted on a commercial dairy farm with automatic feeding and milking systems. Feeding treatments consisted of two different frequencies, high feed delivery frequency (11 deliveries per day) and low feed delivery frequency (six deliveries per day). Lying behavior of 20 dairy cows was electronically monitored. The results obtained showed that 11 deliveries per day feed delivery frequency decreases the number of long-duration lying bouts, which may indicate that a very high feeding frequency disturbs the cows during their resting periods and thus influences both animal comfort and milk production. High feeding frequency may disturb the duration of lying bouts and alter the pattern of lying behavior throughout the day, affecting mainly the lying time during the 60 min before and following the provision of fresh feed. Delivering feed at a low frequency allow cows to distribute more evenly their lying time over the course of the day and improve their utilization of an AMS.

## 1. Introduction

Feeding system in modern dairy farms is an important issue in relation to animal welfare; moreover, the choice between the different types available on the market implies economic and technological consideration. The cost of feed needed for high-yielding herds makes efficient utilization of it essential for profitable farms. Feeding a total mixed ration (TMR) diet is now a preferred practice and has affected the popularity of mechanized feeding systems, mostly represented by conventional manually operated mixer-feeder wagons. The delivery of feed stimulates dairy cows to feed [1,2]. Delivery of TMR in conventional feeding schedules of lactating dairy cattle for most dairy operations remains twice per day (2×). However, many farmers elect to feed their cows only once per day to minimize labor cost. Recently developed automatic feeding systems (AFS) for TMR make it easy to distribute rations frequently and manage feed intake, stimulate cow activity, reduce leftovers, adapt the volume of ration to the size of the animal group, reduce of total energy consumption for feed distribution, and reduce labor costs [3,4]. 

Many researchers have examined the effect of feeding frequency on the performance of dairy cows, mainly on dry matter intake (DMI) and milk production [5,6]. Feeding frequency strongly influences feeding behavior [7]. DeVries et al. [8] showed that increasing the frequency of feed delivery (from 1× to 2× and from 2× to 4×) allowed cows to increase their daily feeding time over the course of the day. Crossley et al. [9] found that increasing TMR delivery frequency from 2× to 6× led to consumption of shorter, smaller meals during peak periods of feed consumption. Lying is one of the most important behaviors performed by dairy cattle. Cows show preference for lying over feeding and social contact when subject to various time constraints, thus feeding frequency is affecting lying behavior [10]. 

Mäntysaari et al. [5] found that cows fed five times a day increased restlessness and decreased lying time compared to cows fed once a day. In contrast, DeVries et al. [8] and Hart et al. [11] found that there was no change in total daily lying time with increased frequency of feed delivery, but the daily distribution of lying time was influenced. Measures of lying behavior are important indicators of cow comfort, providing valuable information on how cows interact with their environment. Total lying time [12,13], number of lying bouts, and bout duration [14] were identified as sensitive measures of stall comfort and have been evaluated as appropriate welfare indicators for lactating dairy cows [13]. Lying behavior in free-stall barns is affected by design and management factors [15,16,17], including milking and feeding management [7,10,18]. Studies have measured lying behavior automatically over a few days by using data loggers [14,19], through time-lapse video [12] or by a computer vision-based system [20]. Electronic data loggers are widely available and can be used to measure lying behavior accurately, including the total time spent lying down, the number of lying bouts, and the duration of each bout for cows [21].

Automatic milking systems (AMS) provide individual cows the freedom to choose their own milking times. Motivation to eat is a stronger incentive for attracting cows to a milking unit than is the motivation to be milked [22,23]. Oostra et al. [24] found no effect of feeding frequency on the daily number of visits to an AMS; however, an increase in frequency (from 2× to 6×) had a positive effect on the utilization of the cowshed facilities, such as the occupation of the feeding fence, cubicles, and feed alley. AMS utilization patterns appear closely linked with feeding patterns. Management practices that distribute feeding bouts evenly throughout 24 h are likely to positively impact AMS utilization levels [22].

To date, the majority of studies that have examined the effect of feeding frequency on cow behavior and performance have taken place in conventional feeding systems in combination with parlor-milking systems. The range of the feeding frequencies in these studies has been between 1× and 5×, and with cows being milked in a conventional parlor at frequencies of 2× and 3×. However, an AFS enables more frequent delivery of fresh feed compared to conventional feeding systems, and in combination with an AMS, provides cows more freedom to determine individual critical behavioral activities (feeding, lying, and milking).

The objective of this study was to determine whether the frequency of feed delivery in an AFS affects: (a) the behavior patterns (lying duration, number of lying bouts, and distribution of lying activity in the day) of dairy cows, (b) visits to an AMS during the day (i.e., percentage of utilization, visiting pattern), and (c) milk yields. We hypothesized that under AFS and AMS, an increased frequency of feed delivery would negatively influence lying behavior. We further hypothesized that the frequency of feed delivery would affect the pattern of lying behavior, particularly after fresh feed delivery and the utilization of AMS.

## 2. Materials and Methods

### 2.1. Animals, Housing and Feed

The study was conducted between December and January on a commercial dairy farm located in Friesland (The Netherlands) where animals were milked with an AMS (DeLaval VMS, DeLaval International AB, Tumba, Sweden) and fed by an AFS (Mix Feeder, Skiold Mullerup, Ullerslev, Denmark).

Two sensors with integrated data loggers, located in the middle of the barn, were used to measure and record the air temperature and relative humidity (HOBO U12 Temp/RH/Light/External Data Logger, Onset Computer Corporation, Pocasset, MA). The average temperatures of the two periods during the experiments were 5.2 °C and 7.9 °C, respectively.

**Figure 1:**
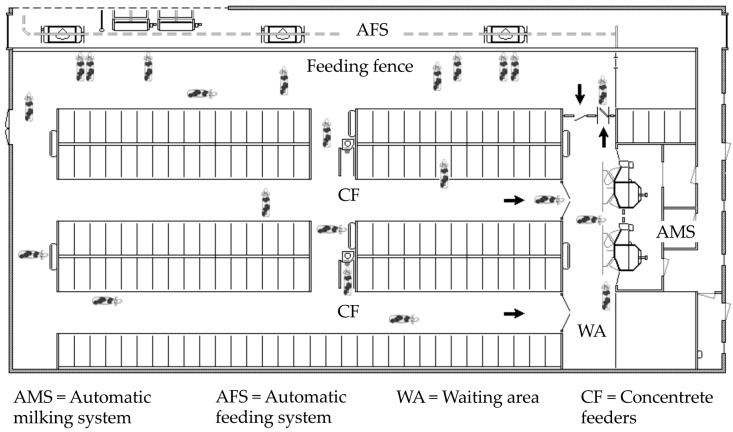
Barn layout with the position of the milking area composed by two automatic milking system units, automatic feeding system, and two automatic concentrate feeders.

The barn housed 93 lactating Holstein Friesian cows, of which 21 were primiparous and 72 were multiparous (at the beginning of the study 2.7 ± 1.5 parity, 138.3 ± 111.5 days in milk (DIM); mean ± SD), and featured a loose-housing layout with a total of 141 cubicles with rubber mats covered with sawdust. The manger, positioned sideways in the barn, had 61 feeding spaces (Figure 1). Twenty lactating cows, of which five were primiparous and 15 were multiparous (3.0 ± 1.7 parity, 200.9 ± 132.2 DIM at the beginning of the data collection period) were randomly selected for behavioral tracking. The milking area consisted of two AMS units and a nearby waiting area in front of the unit entrance. One-way gates assured selectively guided cow traffic, and the animals had access to two AMS units 24 h/day, except at times for system cleaning (three times a day at 04:00, 11:00, and 20:00). The farm fetched the cows with more than 12 h since last milking two times a day, and the minimum time interval between two milkings (6–12 h) was a function of milk yield per cow. All cows were fed one mixed ration (MR) (36.3 kg/day per cow) and concentrates were supplied in the AMS and by two automatic concentrate feeders, placed in the central passage of the lying area (Figure 1). The amount of concentrate (on average 2 ± 0.35 kg/day per cow) was a function of the milk production, DIM, and parity of the cows. The MR consisted of 70.5% grass silage, 23.3% corn silage, 2.4% rape straw, 3.7% soybean meal, and 0.1% minerals, as well as vitamin mix on a DM basis. The daily orts averaged 5.4 ± 3.2% (mean ± SD) of the delivered feed provided over the duration of the experiment.

### 2.2. Experimental Treatments

Treatments consisted of two different frequencies of MR distributions applied to all 93 cows. Each treatment lasted 7 d, in which a 3 d adjustment period [8] was followed by 4 d of data collection on the treatment effects [19,25], replicated in two experimental periods.

**Figure 2:**
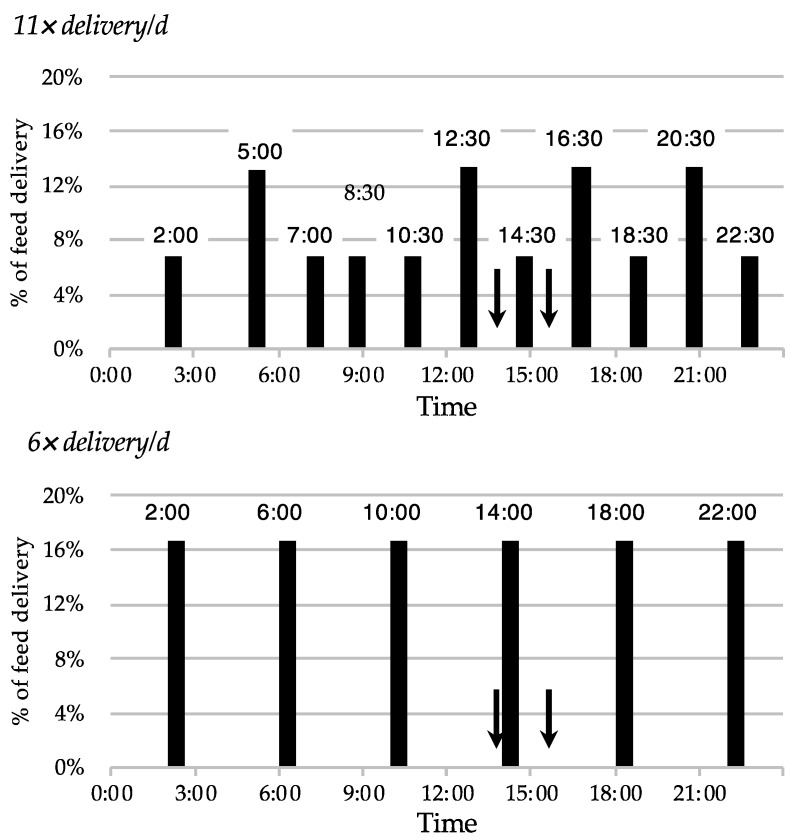
Feeding timetables with time of each delivery and quantity of feed delivered in percentage of MR. Bold arrows indicate times at which the feed was pushed up during the day (13:45 and 15:45).

The two treatments were (1) a feed delivery frequency of 11 deliveries per day (11×) (at 02:00, 05:00, 07:00, 08:30, 10:30, 12:30, 14:30, 16:30, 18:30, 20:30 and 22:30) and (2) a feed delivery frequency of six deliveries per day (6×) (at 02:00, 06:00, 10:00, 14:00, 18:00 and 22:00). In each treatment, feed was pushed up at 13:45 and 15:45 by the AFS (Figure 2). In this study we used a 11× feeding frequency because this was the option used by the farmer, and we chose a 6× feeding frequency because lower feed delivery can be used with the AFS (the mixer wagon has a limited volume and can deliver a limited amount of feed). The aim was to check the response of the cows to a different feeding frequency using the farmer’s settings of the equipment.

### 2.3. Behavioral Recording

Lying behavior patterns of the cows were automatically recorded using two types of electronic data loggers. HOBO Pendant G Data Loggers (Onset Computer Corporation, Pocasset, MA) were utilized to measure the leg orientation at 1 min intervals and allowed all the lying behavior data to be collected electronically [21]. The devices were attached to the lateral side of the right hind leg of each cow by using veterinary bandaging tape (Vet-flex, Kruuse group, Langeskov, Denmark) in a position such that the *x*-axis of the data logger was perpendicular to the ground. The degree of vertical tilt of the *x*- and *z*-axis was used to determine the lying behavior of the animal [21].

IceTag Activity Sensors (v. 2.004, IceRobotics Ltd., Edinburgh, UK) were used to sample acceleration with a frequency of 8 Hz, and to determine the percentage of time the cows spent lying for each recorded second [21]. IceTag sensors were attached to the lateral side of the right hind leg above the fetlock by means of a strap with a buckle. Cow behavior was classified as “lying” or “not lying” for each recorded datum based on the IceTag-recorded intensity thresholds for lying [21]. Finally, data were edited with a filter to remove the effect of short, potentially erroneous readings of lying events. A previous study had shown that these two types of loggers gave the same results and have an agreement of more than 99% with video recordings [21].

Data collected by the data loggers were used to calculate lying times (h/d), bout frequency (n/d), and bout duration (min/bout) for each cow and each day of the treatment. Pre-feeding and post-feeding lying times (min) were calculated as the average lying time per cow during the 60 min before and following provision of each fresh feed delivery (feeder wagon starts automatic feed distribution). These two 60 min periods were identified in an explorative analysis as the times when lying behavior of cows was especially influenced by feed delivery [7]. The pre-feeding and post-feeding periods were determined with reference to the time of deliver and assessing the lying time 60 min before and 60 min after that time for both feeding frequency and each feed delivery. The behavioral observation period covered the days of treatment (4 d) for each feed delivery frequency in each period for a total of 16 d.

### 2.4. Cow Visits to the AMS

Milking-related data for all 93 cows, including time of entrance to and of exit from the AMS, yield and type of visit per visit, were automatically collected by the AMS and stored as “log files”. AMS log files included classifications for three types of cow visits: milkings (i.e., the cow was milked normally), refusals (i.e., the cow had no permission to be milked), and failures (i.e., the milking was not completed normally). The milking duration (time in AMS) was calculated as the difference between the times that a cow entered and exited the AMS. The log files were preprocessed with the support of MS-Excel 2007, and the mean milk yield (kg/d), milking frequency (n/d), milking duration (min/m), refusal frequency (n/d), and visit frequency (n/d) on a per cow basis were calculated for each day of each feed delivery treatment. The average hourly number of milkings and AMS refusals per cow were calculated for each feed delivery treatment.

### 2.5. Statistical Analysis

Descriptive statistics were used to characterize the distribution of the variables in the study using the MEANS and FREQ procedures of SAS^®^ software (Copyright^©^ 2008, SAS Institute Inc. SAS and all other SAS Institute Inc. product or service names are registered trademarks or trademarks of SAS Institute Inc., Cary, NC, USA). Before analyses, all data were screened for normality using the UNIVARIATE procedure of SAS^®^ software. Only lying behavioral residuals were transformed using logarithmic transformations (log10(x)) to achieve normal distributions. Lying behavioral data (lying times, bout frequency, bout duration, and pre-feeding and post-feeding times), AMS utilization (milking frequency and duration, refusal frequency, and visit frequency), and milk yield were analyzed as repeated measures using the MIXED Procedure of SAS^®^ software for testing the effects of feeding frequency treatment (11× and 6× feed delivery), period, and the interaction between feeding frequency and period. The cows monitored were considered replicates as their behavior can be considered independent. In fact, synchronized behavior has not been observed as the daily average percentage of animals simultaneously exhibiting the same posture (lying or standing) was 50.8% and lower than the threshold percentage of 70% for 90% of time [26]. Least squares means and standard errors were determined using the LSMEANS and STDERR statement in PROC GLM of SAS^®^ software. The replicate was considered a random effect and cow within time was a subject effect. The fixed effects of type of treatment (11× and 6× feed delivery), period (Period 1 vs. Period 2), and their interaction (Treatment × Period) were included in the model, fitting a Kenward-Roger method to calculate the denominator degrees of freedom to approximate the F-tests in the mixed models.

The effect of feeding frequency on distribution of lying bouts duration were analyzed using the GLM procedure of SAS^®^ software with the LSMEANS options. Five subsets of bout duration were used: the first contained all short lying bouts with duration <50 min; the second contained only lying bouts with a duration between 50 min and 100 min; the third subset contained the lying bouts with a duration between 100 min and 150 min; the fourth contained the bouts with a duration between 150 and 200 min; and the last subset contained all long bouts with a duration >200 min.

The effect of the feeding frequency on the diurnal AMS visiting pattern (milkings and refusals) was assessed using a model including the following fixed factors: treatment (11× and 6× feed delivery), hour (1 to 24) and treatment × hour interaction.

In the statistical analyses, significance was declared when *p* < 0.05 and *p* < 0.01 (* *p* < 0.05; ** *p* < 0.01). A tendency was declared when *p* < 0.10.

## 3. Results

The frequency of feed delivery had no significant correlation with total daily lying time, lying bout frequency and bout duration (Table 1). However, the frequency of feed delivery did affect the pattern of lying behavior throughout the day and modified the average lying time per cow during the 60 min before and following provision of feed. Feed delivery 11 times a day compared to six times a day significantly decreased pre-feeding lying time (28.88 min vs. 32.77 min per cow, respectively; *p* = 0.01) and increased post-feeding lying time (30.84 min vs. 26.85 min per cow, respectively; *p* = 0.03). Furthermore, we observed effect of period on the length of time cows spent lying per day. In period two cows increased their lying time of about one hour (from 11.6 h to 12.8 h per day).

**Figure 3:**
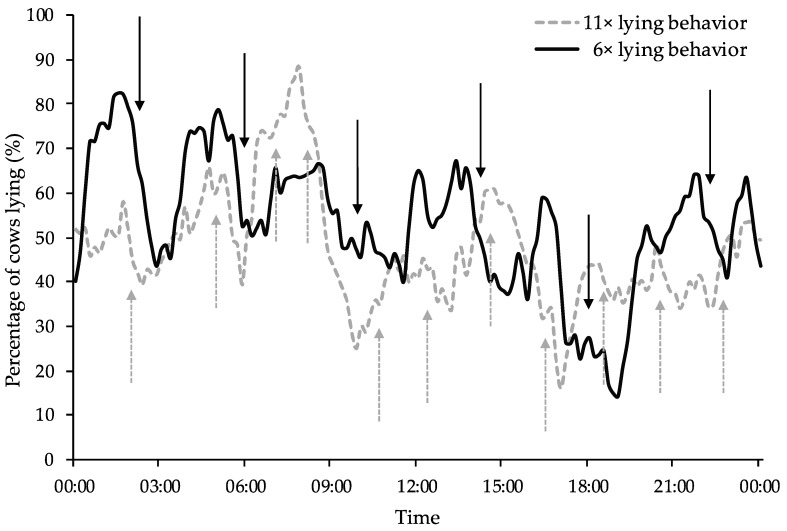
Percentage of cows lying down over a 24 h period (percentage for each 10 min mean interval during the day). Cows were monitored under two feed delivery frequencies (11× and 6×). Data are averaged for 4 d for each treatment and period. Solid black arrows indicate times at which the cows were fed 6× and the dashed grey arrows indicate times at which the cows were fed 11×.

The effect of feed delivery is also visible in Figure 3 which shows the distribution of the percentage of cows lying down over a day.

When the cows were fed 11 times a day, the percentage of cows lying was below 30% twice; compared to only once when they were fed 6×. The differences in lying behavior between treatments were particularly pronounced at times of peak feeding behavior (03:00, 06:00, 11:00, 15:00, 18:30 and 22:30) after each delivery; lying activity during the period immediately after the distribution of fresh feed decreased an average of 30% when cows were fed at low frequency (6×). In fact, when the cows were fed 6×, fewer cows were lying during the time period immediately following the provision of fresh feed, as highlighted by post-feeding lying time. Cows fed 6× showed a decrease in percentage of lying behavior after each feeding, whereas the 11× feed delivery did not show this trend. The peak for 11× lying behavior appears to be higher than any peak for 6×, and lying behavior of cows fed 6× appeared to spend more time (<30%) than 11×, even if 11× cows reach below 30% more times. Also, the lying pattern appears to be similar among the treatment, especially during the late evening or night and early morning.

**Figure 4:**
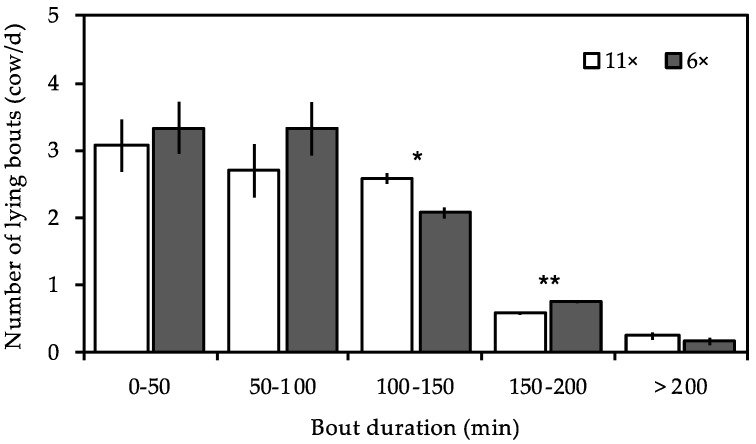
Distribution of lying bouts duration. Cows were monitored under two feed delivery frequencies (11× and 6×). Data are the average of the two periods for each feed delivery frequency. The error bars represent ± 1 SE of the mean. Statistical differences between feed delivery are indicated with **(*p* < 0.01) and *(*p* < 0.05).

Figure 4 shows the effect of feeding frequency on distribution of lying bouts duration. We observed a difference in the number of lying bouts that had durations between 100 min and 200 min. Cows fed 11 times a day compared to those fed six times a day had significantly fewer lying bouts lasting between 150 min and 200 min (*p* < 0.01), but significantly more lying bouts with a duration between 100 min and 150 min (*p* < 0.05).

During period 2, bout frequency was increased respect the period 1 (Table 1). Also, the utilization of the AMS (visit frequency) during period 2 increased, in particular by an increase of refusal frequency. We found no effect of feed delivery frequency on milking frequency, refusal frequency, or visit frequency (Table 1). Instead, we observed effect of treatment on milking duration.

**Figure 5:**
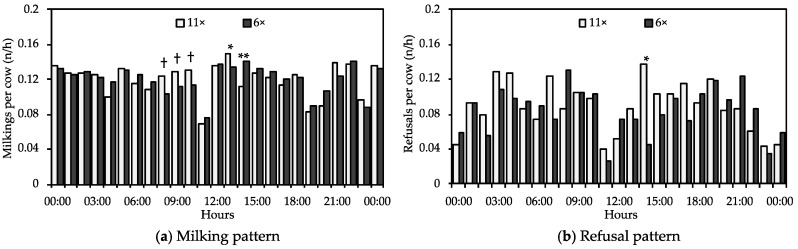
Average hourly (**a**) number of milkings and (**b**) automatic milking system (AMS) refusals per cow for different feed delivery frequencies (11× and 6×). Data are the average of the two periods for each feed delivery frequency. Statistical differences between feed delivery are indicated with **(*p* < 0.01) and * *p* < 0.05). A tendency is indicated with † (*p* < 0.10).

We did find that the feeding frequency affected the diurnal AMS visiting pattern. To better analyze the effect the total visits have been divided into milkings and refusals (Figure 5). The number of milkings per cow per hour was similar for most hours of the day for both treatments, but the high feeding frequency (11×) tended (*p* < 0.10) to increase the number of milkings during the morning, between 08:00 and 10:00, caused a significant increase at 13:00 (*p* < 0.05), and resulted in a significantly lower number of milkings at 14:00 (*p* < 0.01) compared to the low feeding frequency (6×). The high feeding frequency led to significantly more refusals per cow per hour only at 14:00 (*p* < 0.05) compared to the low feed frequency.

The low feeding frequency showed a significant effect to result in a higher milk yield (*p* = 0.01, Table 1). The average milk yield was 31.32 and 32.15 kg/d per cow for the 11× and 6× treatments, respectively.

## 4. Discussion

In the current study, cows spent, on average, 12.2 h/d lying down. These values are comparable to values ranging from 11.0 to 11.9 h/d reported in the literature for conventional milking parlors [19,27] and to a lying duration of 11.2 ± 2.5 h/d observed by DeVries et al. [28] and Deming et al. [29] in an AMS herd. A change in feeding frequency did not result in a change in daily lying time. Lying behavior is relatively consistent among dairy cows, which tend to modify other behaviors to conserve lying time [9]. Our results correspond with those reported in previous studies [8,9,11], which show that an increased frequency of feed delivery (from 1× to 2× and from 2× to 4×; from 2× to 6×; from 1× to 3×) did not affect the total daily lying time. It should be noticed, however, that these studies used a relative low feed delivery frequency compared to our studies. Nevertheless, we might expect a reduction of the lying time as the studies by Mäntysaari et al. [5] on increasing feeding frequency from one to five times a day resulted in a decreased daily lying time. It should be considered that our adaptation periods (of 3 d) might hide a full response to treatment, although has been used by other authors. Other authors suggest that adaptation periods of 7 d to 14 d are more suitable to detect a response to treatment [30]. 

We observed that the frequency of feed delivery affected the pattern of lying behavior throughout the day (daily distribution of lying time) and the lying time during the 60 min before and following the provision of fresh feed. These patterns could reflect the periods of higher feeding activity throughout the day of the cows, which were related to feed delivery times. Cows spent 12% more time lying during the hour before feed delivery and 13% less time lying in the hour following feed delivery when provided feed 6× compared with feed 11×. This displacement in lying time during the 60 min before and following the provision of fresh feed indicates that the delivery of fresh feed is a much stronger stimulus to get cows to feed, especially when cows are fed with high frequency. However, these results demonstrate that the cows are able to adjust and change their behavior against variation of feeding frequency and evenly distribute their lying time over the course of the day, on basis of feeding frequency delivery. Also, in the studies by DeVries and von Keyserlingk [7] and DeVries et al. [8], the daily distribution of lying time was influenced by frequency of feed delivery and feeding times. In our study, the change in daily distribution of lying time in response to decreased frequency of feed delivery resulted in an increased percentage of cows lying down during the late evening and early morning hours (21:00 to 06:00), as well as during mid-day (09:00 to 14:00). This result agrees with the findings of Phillips and Rind [6] and DeVries et al. [7], who reported that cows fed frequently tended to spend a longer time feeding in the late evening. The daily lying pattern is correlated with feeding frequency and demonstrates that the cows distribute their lying time evenly over the course of the day in relation to delivery of feed (frequency and delivery times).

In this study, we observed that the feeding frequency affected the distribution of lying bouts durations. The feeding frequency affected the longer lying bouts (those lasting between 100 min to 200 min), but not the shorter bouts (those less than 100 min). The possible explanation may be that cows fed 6× are motivated to feed less frequently and thus have more time to lie down continuously between deliveries of fresh feed, as demonstrated by the fact that lying has priority over feeding [10]. In contrast to cows fed 6×, cows fed 11× interrupted their longer lying bouts (>150 min) because there was less time between feed deliveries but increase the lying bouts with a duration of 100–150 min, which is compatible with the time between two feed deliveries.

Feeding frequency had no effect on the milking, refusal and visit frequency. However, during several hours of the day, the feeding frequency significantly affected the milking and refusal patterns. Our results for the effect of the feeding system on the daily number of milkings and refusals are consistent with those of Oostra et al. [24] and Belle et al. [3], who concluded that the feeding frequency had no significant effect on the number of daily milkings and refusals per cow. The frequency of feeding is not the only action that influences the visiting pattern of dairy cows to the AMS: cleaning the milking robot [23], management factors like stocking density at the AMS (cows/AMS) [22,29], and animal factors like fetching cows and herd dynamics [22] are other factors that affect this pattern.

In the present study, the lower feeding frequency increase significantly milk yield. In the study by Phillips and Rind [6], increased milk production was observed at a lower feeding frequency and was associated with increased feed intake. These findings are contrary to those of previous research that observed no effect of the frequency of feed delivery on milk yields [9,11].

Automatic milking systems in combination with AFS provide cows more freedom to determine individual moments of lying, feeding, and milking. Compared to conventional dairy systems, where synchronization of behavior does still occur (particularly around times of milking and feed delivery), in AFS and AMS behavioral activity is distributed over a 24 h period. This may explain the subdued effects of feeding frequency found in this study in comparison with other studies that have examined the effect of feeding frequency in conventional feeding systems in combination with parlor-milking production systems.

Neither lying behavior nor utilization of AMS was affected by decreasing the number of daily feed deliveries via AFS in this study. In our study, high feeding frequency (11× feeding treatment) showed significant differences in the pre-feeding and post-feeding lying time, and in the duration of the longer lying bouts, which supports a conclusion that very high feeding frequencies disturb lying behavior throughout the day, affecting mainly the lying time coincident with the provision of fresh feed. The feeding frequency also had a significant effect on the milking and refusal patterns throughout the day. 

## 5. Conclusions

In conclusion, results obtained, may indicate that a very high feeding frequency disturbs the cows during their resting periods and this may influence both animal comfort and milk production. High feeding frequencies should be avoided to allow cows to distribute both their lying time and their utilization of AMS more evenly over the course of the day. So, it is necessary to find a feeding frequency that is high enough to distribute the feeding time of cows over the course of the day, yet not so high that it disturbs the pattern of lying behavior and long-duration lying bouts. Further studies concerning feeding places, feeding and aggressive behaviors, and individual feed intake in farms with AFS would be useful to help explain some of the other associations we observed and to improve feeding management and the productivity and comfort of lactating dairy cows.

## Figures and Tables

**Table 1:** Effect of feeding frequency, period, and interaction between feeding frequency and period on lying behavior, utilization of the automated milking system (AMS), and milk yield (least-squares means).

Parameters	Period 1		Period 2		Effect ^3^			
	Feeding Frequency							
Lying Behavior	11× ^1^	6× ^1^	11× ^1^	6× ^1^	SE ^2^	F	Pe	F × Pe
Lying time (h/d)	11.39	11.75	12.87	12.75	0.22	0.59	<0.001	0.28
Bout frequency (n/d)	8.53	8.75	9.75	10.56	0.51	0.31	0.01	0.56
Bout duration (min/bout)	85.08	84.27	91.57	82.76	4.35	0.27	0.56	0.36
Pre-feeding lying (min)	28.21	31.13	29.54	34.41	1.53	0.01	0.13	0.52
Post-feeding lying (min)	30.95	26.86	30.74	26.83	1.83	0.03	0.95	0.96
**Utilization of AMS**								
Milking frequency (n/d)	2.84	2.78	2.84	2.92	0.03	0.82	0.02	0.01
Milking duration (min/m)	7.69	7.92	7.25	7.23	0.05	0.02	<0.001	0.01
Refusal frequency (n/d)	1.74	1.37	2.71	2.69	0.17	0.24	<0.001	0.30
Visit frequency (n/d)	4.59	4.15	5.55	5.61	0.17	0.28	<0.001	0.16
Milk yield (kg/d)	31.78	32.32	30.85	31.97	0.30	0.01	0.03	0.33

^1^ Frequency: 11× = feed delivery 11 times a day at 02:00, 05:00, 07:00, 08:30, 10:30, 12:30, 14:30, 16:30, 18:30, 20:30 and 22:30; 6× = feed delivery six times a day at 02:00, 06:00, 10:00, 14:00, 18:00 and 22:00. ^2^ SE = standard error. ^3^ F = frequency of feed delivery; Pe = period; F × Pe = interaction.
